# Prolonged Retention of the Placenta

**Published:** 1898-09-24

**Authors:** 


### PROLONGED RETENTION OF THE PLACENTA.

As a thing to note and remember, Dr. Wiggins puts on record[1] a case in which a woman had eight abortions, in all of which, with one exception, the placenta was retained for periods varying from two to six weeks. She never went beyond the fifth month of pregnancy, and usually made a rapid recovery. She was strong, and well built, and there was no history of syphilis. The last abortion took place on June 24th, and the placenta was expelled by natural pains on July 11th; the membranes were degenerated, but the placenta was fresh and well formed, and no odour of decomposition could be detected. Curiously the mammary glands continued flaccid long after the expulsion of the fœtus, the milk appearing on the third day after the placenta was expelled, when the quantity became abundant—a condition which had occurred in all the eight miscarriages.

[1] Brit. Med. Jour., Sept. 10.

